# Nitric Oxide‐Releasing Bioinspired Scaffold for Exquisite Regeneration of Osteoporotic Bone via Regulation of Homeostasis

**DOI:** 10.1002/advs.202205336

**Published:** 2022-12-29

**Authors:** Jun‐Kyu Lee, Da‐Seul Kim, So‐Yeon Park, Seung‐Woon Baek, Ji‐Won Jung, Tae‐Hyung Kim, Dong Keun Han

**Affiliations:** ^1^ Department of Biomedical Science CHA University 335 Pangyo‐ro, Bundang‐gu, Seongnam‐si Gyeonggi‐do 13488 Republic of Korea; ^2^ School of Integrative Engineering Chung‐Ang University 84 Heukseok‐ro, Dongjak‐gu Seoul 06974 Republic of Korea; ^3^ Division of Biotechnology College of Life Sciences and Biotechnology Korea University Seongbuk‐gu Seoul 02841 Republic of Korea; ^4^ Department of Biomedical Engineering SKKU Institute for Convergence Sungkyunkwan University (SKKU) 2066 Seobu‐ro, Jangan‐gu, Suwon‐si Gyeonggi‐do 16419 Republic of Korea; ^5^ Department of Intelligent Precision Healthcare Convergence SKKU Institute for Convergence Sungkyunkwan University (SKKU) 2066 Seobu‐ro, Jangan‐gu, Suwon‐si Gyeonggi‐do 16419 Republic of Korea

**Keywords:** alendronate, bioinspired scaffold, bone homeostasis, bone morphogenetic protein 2, bone regeneration, osteoporosis, zinc oxide

## Abstract

Osteoporotic bone regeneration is a challenging process which involves the occurrence of sophisticated interactions. Although various polymeric scaffolds have been proposed for bone repair, research on osteoporotic bone regeneration remains practically limited. In particular, achieving satisfactory bone regeneration when using osteoporotic drugs is challenging including bisphosphonates. Here, a novel nitric oxide‐releasing bioinspired scaffold with bioactive agents for the exquisite regeneration of osteoporotic bone is proposed. The bone‐like biomimetic poly(lactic‐co‐glycolic acid) scaffold is first prepared in combination with organic/inorganic ECM and magnesium hydroxide as the base implant material. Nanoparticles containing bioactive agents of zinc oxide (ZO), alendronate, and BMP2 are incorporated to the biomimetic scaffold to impart multifunctionality such as anti‐inflammation, angiogenesis, anti‐osteoclastogenesis, and bone regeneration. Especially, nitric oxide (NO) generated from ZO stimulates the activity of cGMP and protein kinase G; in addition, ZO downregulates the RANKL/osteoprotegerin ratio by suppressing the Wnt/*β*‐catenin signaling pathway. The new bone is formed much better in the osteoporotic rat model than in the normal model through the regulation of bone homeostasis via the scaffold. These synergistic effects suggest that such a bioinspired scaffold could be a comprehensive way to regenerate exceptionally osteoporotic bones.

## Graphical abstract

An advanced strategy of the preparation for bone graft material using biomimetic scaffold is proposed. Functional nanoparticle which consists of three bioactive molecules demonstrates outstanding angiogenic and osteogenic effects. The nanoparticle‐immobilized bioinspired scaffold exhibits multifunctional activity in anti‐inflammation, angiogenesis, anti‐osteoclastogenesis, and bone regeneration.

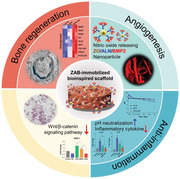

## 1 Introduction

Bone is a rigid tissue that undergoes constant remodeling throughout an individual's life. However, osteoporosis (OP) can make the hard tissue fragile and brittle by breaking the homeostasis between osteoblasts and osteoclasts.^[^ ^1^ ^]^ OP can occur in any condition, but it is an age‐related disease that more frequently appears in women than men, particularly when menopause begins or ovaries are removed.^[^ ^2^ ^]^ Compared to healthy individuals, patients with OP experience a higher risk of bone fracture and an extended healing period. Therefore, the regeneration of critical‐sized bone fractures in osteoporosis involves various challenges in clinical trial applications. Lack of adequate research on the regeneration of bones in harsh environments such as OP points to the need for a multifunctional scaffold for bone regeneration. When the bone fracture occurs, complex bone regenerative processes such as inflammation, soft callus formation, and bone remodeling are initiated. Although many existing strategies are focused on targeting osteoblast lineage cells to stimulate osteogenesis, the ideal scaffold should have suitable abilities, such as biocompatibility, osteoconductivity, osteoinductivity, anti‐inflammatory activity, angiogenesis, and inhibition of excessive osteoclastogenesis for complete bone tissue regeneration.^[^ ^3^ ^]^

The FDA approved poly(lactic‐co‐glycolic acid) (PLGA) for several bone implants because of its biodegradability and biocompatibility. However, it has been reported that degradation of PLGA generates acidic monomers, lactic acid and glycolic acid, and these byproducts cause an acidic microenvironment at the implant site.^[^ ^4^ ^]^ In our previous studies, the magnesium hydroxide (MH) exhibited superior pH neutralizing ability for diverse tissue regeneration.^[^ ^5^ ^]^ Since inorganic molecules are generally difficult to disperse in an organic solvent, ricinoleic acid was used to provide dispersibility for MH. The modified MH (mMH) was easy to utilize for the scaffold fabrication and reduced the inflammatory response. Extracellular matrix (ECM) generally provides various effects, such as direct cell adhesion, spreading, migration, and differentiation.^[^ ^6^ ^]^ The bovine‐derived decellularized bone extracellular matrix (bECM, inorganic ECM) and demineralized bone matrix (DBM, organic ECM) were combined in a ratio of 7:3 to construct the bone‐mimicking environment in the porous scaffold. The combination of both ECMs simultaneously imparted scaffold osteoconductivity and osteoinductivity. The base material for the bone implant was this biomimetic PLGA/mMH/ECM scaffold (BPM).

Some materials, such as metals and metal oxides, have been reported to mimic the natural catalytic enzymes.^[^ ^7^ ^]^ McCarthy et al. demonstrated that the various transition metals could generate NO with *S*‐nitroso‐*N*‐acetylpenicillamine (SNAP) under physiological pH conditions.^[^ ^8^ ^]^ We used ZO nanoparticles as the glutathione peroxidase and glycosidase to sustainedly release NO on the BPM. NO is an endogenously synthesized signaling molecule produced in endothelial cells by stimulated nitric oxide synthase. As a result, with an increase in PKG expression levels, sustained release of NO can influence angiogenesis, vascular remodeling,^[^ ^9^ ^]^ vasodilation, and blood flow in the cardiovascular system.^[^ ^10^ ^]^

Alendronate (ALN), an amino‐bisphosphonate, is one of the most frequently used drugs to treat OP.^[^ ^11^ ^]^ The nitrogen‐contained bisphosphonates affect differentiation and apoptosis of osteoclast by downregulating farnesyl pyrophosphate synthase (FPPS), an intermediate in the mevalonate pathway of sterol biosynthesis.^[^ ^12^ ^]^ The prenylation added hydrophobic molecules to substrates with FPPS that synthesized small GTPases such as Rap, Ras, Rac, and Rho. In contrast, the unprenylated GTPases were accumulated in the cytoplasm of the osteoclast.^[^ ^13^ ^]^ Consequently, the absorbed bisphosphonates induce apoptosis of osteoclasts and inhibit bone resorption.^[^ ^14^ ^]^

**Figure 1:**
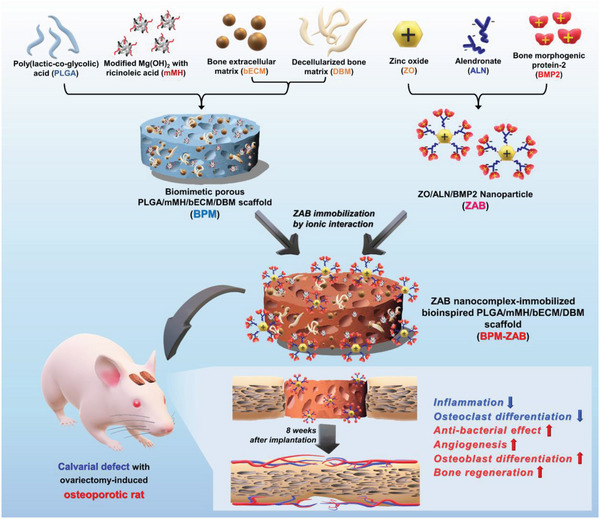
Schematic illustration of the ZAB‐immobilized bioinspired polymeric scaffold. Illustration of the composition of bioinspired porous PLGA/mMH/bECM/DBM scaffold (BPM) that was prepared as a base material. The ZO/ALN/BMP2 nanoparticles (ZAB) were immobilized in a supersaturated Ca/P solution on the BPM (BPM‐ZAB) by ionic interaction. The BPM‐ZAB can effectively restore critical‐sized bone defects in ovariectomy‐induced rats with osteoporosis with its multifunctional regenerative effects: attenuating inflammation and osteoclast differentiation and enhancing antibacterial effect, angiogenesis, osteoblast differentiation, and bone regeneration.

The recombinant human bone morphogenetic protein 2 (BMP2) has been used for orthopedic surgery for a few decades.^[^ ^15^ ^]^ However, it has been reported that diverse side effects, such as ectopic bone formation, osteoclast‐mediated bone resorption, and inappropriate adipogenesis, may occur if BMP2 is utilized without a method that can induce sustained release.^[^ ^16^ ^]^ A combination of the ZO/ALN/BMP2 nanoparticles (ZAB) is prepared to induce the sustained release of NO, ALN, and BMP2, respectively. In this study, we prepared a ZAB‐immobilized bioinspired PLGA/MH/ECM scaffold (BPM‐ZAB) using supersaturated‐calcium solution (**Figure** **1**). We demonstrate that the BPM‐ZAB could serve as a promising biodegradable bone implant for effective bone healing in rats with OP.

## 2 Results

### 2.1 Characteristics of ZAB Nanoparticles and ZAB‐Immobilized Bioinspired Scaffold

**Figure 2:**
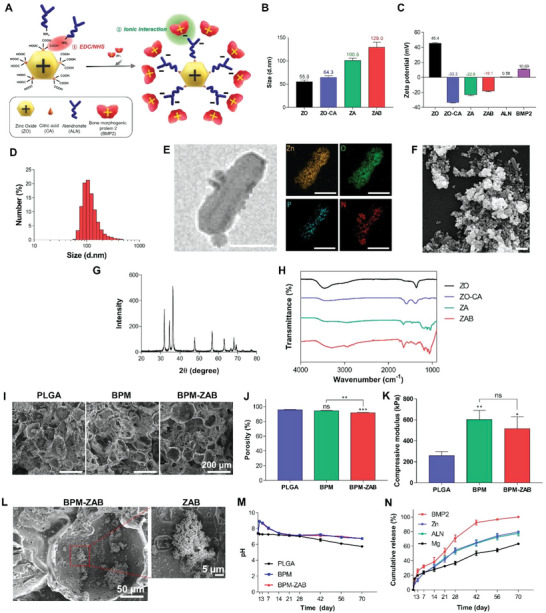
Characterization of ZAB and ZAB‐immobilized bioinspired PLGA/MH scaffolds. A) A schematic representation of the synthesis of ZAB. Data measured by dynamic light scattering (DLS; *n* = 3): B) The hydrodynamic size of nanoparticles according to the complexation of ALN and BMP2. C) Zeta potential analysis of ZAB after binding with various materials: ZO, zinc oxide; ZO‐CA, ZO modified with citric acid; ZA, zinc oxide modified with citric acid and ALN; and ZAB, zinc oxide modified with ALN and BMP2. D) The size distribution of ZAB. E) High resolution‐transmission electron microscopy (HR‐TEM) images of ZAB with energy dispersive X‐ray spectroscopy (EDS) for elemental mapping (scale bar, 100 nm). F) Field emission‐scanning electron microscopy (FE‐SEM) images of ZAB (scale bar, 100 nm). G) Diffraction pattern observed from powder X‐ray diffraction (PXRD). H) Attenuated total reflectance‐Fourier transform‐infrared spectroscopy (ATR‐FTIR) spectra of ZAB. I) Representative SEM image of the PLGA, BPM, and BPM‐ZAB (scale bar, 100 µm). J) The porosity of the scaffolds (*n* = 3). K) Mechanical properties of the scaffolds. Compressive modulus denoting 5–10% compressive stress/strain curves based on stress–strain curve (*n* = 3). L) FE‐SEM image of ZAB on BPM‐ZAB (scale bar, 50 µm). The enlarged image shows the immobilized ZAB crystal (scale bar, 5 µm). M) Changes in the pH value on each scaffold in PBS solution at 37 °C (*n* = 3). N) Cumulative release of the BPM‐ZAB components. bone morphogenetic protein 2 (BMP2; red), zinc ion (Zn; blue), alendronate (ALN; green), and magnesium ion (Mg; black) (*n* = 3). #*p* < 0.0001, ***p < 0.001, **p < 0.01, and *p < 0.05 indicate statistically significant differences, respectively.

ZAB was formed by bonding the carboxylic group of citric acid (CA) on the surface with the amine group of ALN using the EDC/NHS reaction and then bonding the positively charged BMP2 with the negatively charged ZA (**Figure** **2**A). The ZO exhibited 55.0 nm of hydrodynamic size and +45.4 mV of zeta potential (Figure 2B,C). The CA changed the surface charge of ZO to negative. After binding CA, the size increased to 64.3 nm, and the charge reversed to −33.3 mV. The ZO‐ALN (ZA) nanoparticles were complexed with BMP2 by electrostatic interaction between the negative charge of the core particle and the positive charge of BMP2. ZAB displayed a narrow size distribution, and the peak was at 105.7 nm (Figure 2D). For further investigation of ZAB, HR‐TEM/EDS and FE‐SEM were conducted. HR‐TEM/EDS confirmed the microstructure and elemental composition of the ZAB; zinc (Zn, yellow), oxygen (O, green), phosphorous (P, cyan), nitrogen (N, red)) (Figure 2E). ZAB exhibited self‐aggregated shape as a result of dehydration, with a crystalline size of <100 nm (Figure 2F). SEM image display that had a size of ZAB was smaller (≈90 nm) than estimated with dynamic light scattering (DLS) measurements (≈120 nm); this is attributable to the fact that the DLS determines the hydrodynamic size.^[^ ^17^ ^]^ Powder X‐ray diffraction (PXRD) was executed to examine whether ZAB's crystalline structure was changed (Figure 2G and Figure S1, Supporting Information). The ZAB showed hexagonal ZnO wurtzite structure according to miller indices.^[^ ^18^ ^]^ The formation of chemical bonds was confirmed using ATR‐FTIR (Figure 2H).

The PLGA was chosen as the main matrix of the scaffold prepared by the freeze‐drying method. The interconnected microstructure of the scaffolds was observed with SEM (Figure 2I). The porous structure was randomly distributed. The addition of MH, ECMs, and ZAB on the scaffolds did not affect the morphological structure, and their porosity was 95.7%, 94.31%, and 91.79%, respectively (Figure 2J). The compressive strength and modulus also increased with the addition of inorganic molecules (Figure 2K). The compressive modulus of the BPM‐ZAB improved to 516.4 kPa compared to the PLGA with the compressive modulus of 258.7 kPa. No significant difference between BPM and BPM‐ZAB indicated that the immobilization process could not critically affect the mechanical property. The morphology of ZAB and elemental distribution on the scaffold were visualized using FE‐SEM and EDS mapping (Figure 2L and Figure S4, Supporting Information). The images represented that ZAB and calcium phosphate ions were coated onto the scaffold as well as all elements were evenly distributed without aggregation. The amount of each element was determined using an ICP‐OES. It demonstrated that the immobilization could deposit calcium and phosphorous on the scaffold (Table S1, Supporting Information). The additives improved the hydrophilicity of the scaffold (Figure S6, Supporting Information). The scaffolds were immersed in phosphate‐buffered saline (PBS) solution for 10 weeks at 37 °C to verify pH neutralizing capacity (Figure 2M). After 70 days, the pH of the PLGA decreased to about 5.8, whereas in the BPM and BPM‐ZAB, the pH decreased to 6.8. These results imply that the mMH could perform as an effectual pH neutralizing agent in an acidic environment. The 500 µg of ZAB per scaffold was used for immobilized, and the immobilizing efficiency was evaluated to be ≈31%. The release profiles of Zn and ALN exhibited a similar release trend (Figure 2N). The Zn and ALN were released at about 79.5% and 77.5%, respectively, 70 days after degradation. Enzyme‐linked immunosorbent assay (ELISA) was used to determine the loading amount and release of BMP2. The kinetics showed a slightly rapid release in the first 7 days (about 31%), followed by a fully sustained release for 70 days.

### 2.2 Nitric Oxide Generation Ability of BPM‐ZAB

**Figure 3:**
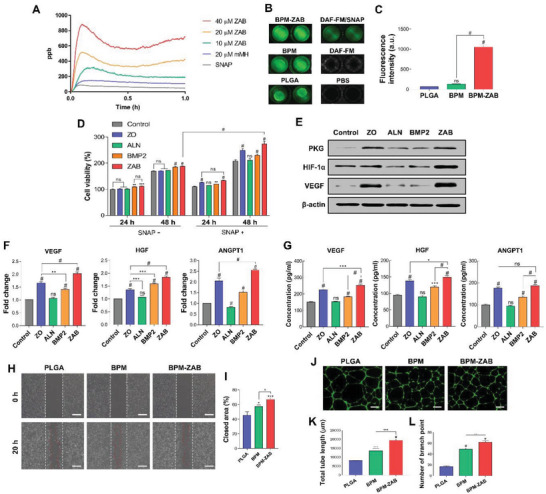
ZAB promotes angiogenesis with NO release. A) NO release profiles with the ZAB and mMH particles using a nitric oxide analyzer (NOA). NO release from each scaffold with SNAP (10 µm): B) The fluorescent images were visualized using NO‐sensitive fluorescent dye, DAF‐FM. C) Quantification of fluorescent intensity. D) The proliferation of human umbilical vein endothelial cells (HUVECs) with or without SNAP for 24 and 48 h (*n* = 3). Angiogenic effect confirmation of the ZAB nanoparticle using HUVECs cultured with SNAP for 48 h: E) Western blot of vascular endothelial growth factor (VEGF), hepatocyte growth factor (HGF), angiopoietin 1 (ANGPT1), protein kinase G (PKG), and hypoxia‐inducible factor 1 alpha (HIF‐1*α*). F) Gene expression levels of angiogenic factors by quantitative PCR with reverse transcription (RT‐qPCR; *n* = 3). G) Protein expressions of angiogenic factors (VEGF, HGF, and ANGPT1) by enzyme‐linked immunosorbent assay (ELISA; *n* = 3). Angiogenic effect confirmation of the BPM‐ZAB scaffold using HUVECs: H) Cell Migration Assay: optical images (scale bar, 200 µm) and I) quantification. J) Tubule‐forming assay (*n* = 3): calcein AM‐stained images (scale bar, 100 µm). K) Quantification of total tube length. L) Quantification of a number of branch point. *#p* < 0.0001, ***p < 0.001, **p < 0.01, and *p < 0.05 indicate statistically significant differences, respectively.

The release of NO through treatment of SNAP at 37 °C using a nitric oxide analyzer (NOA) was performed (**Figure** **3**A) to assess the NO‐releasing ability of the ZAB. The SNAP group showed slight NO release due to catalyzation by the ions in the PBS solution. As the ZAB concentration increased, the release of NO also increased. ZAB displayed a better releasing capacity compared to mMH at equal concentration. ZAB (155 µg) was immobilized on the scaffolds, and the same amount of ZAB was assessed for NO‐releasing capacity using NOA. ZAB exhibited constant NO‐releasing rate (≈600 ppm ≈6.07 nmol L^−1^ [nm]). Furthermore, the low concentration of NO could promote cell survival and proliferation. We examined the NO‐releasing ability of the BPM‐ZAB, and the NO production was imaged and quantified at 6 h using a fluorescence imaging system (Figure 3B,C). The high intensity of the BPM‐ZAB revealed that ZAB effectively enhanced the NO‐generating capacity of the scaffold.

The behaviors were analyzed based on the presence or absence of SNAP on human umbilical vein endothelial cells (HUVECs) to confirm the effects of NO on angiogenesis. Regardless of whether the NO donor was present, ALN showed no difference (Figure 3D). BMP2 exhibited an increase with SNAP at 24 and 48 h, respectively. In contrast, the ZO and ZAB could considerably trigger the proliferation in the presence of NO donors. The expression levels of the angiogenesis‐related markers such as vascular endothelial growth factor (VEGF), hepatocyte growth factor (HGF), and angiopoietin 1 (ANGPT1) were determined by Western blot, qPCR, and ELISA (Figure 3E–G). The ZAB and ZO remarkably enhanced the expression of all angiogenesis‐related molecules, establishing that the sustained release of NO improved vascularization. The migration and vessel generating capacity of ZAB and the scaffolds were evaluated using HUVECs (Figure 3H–L and Figures S9 and S10, Supporting Information). The ZAB increased the closed area, total tube length, and number of branch points, representing the blood vessel network level, compared to the control. These results implied that the BPM‐ZAB has angiogenic effects.

### 2.3 RNA‐Sequencing of hBMSCs on BPM‐ZAB

**Figure 4:**
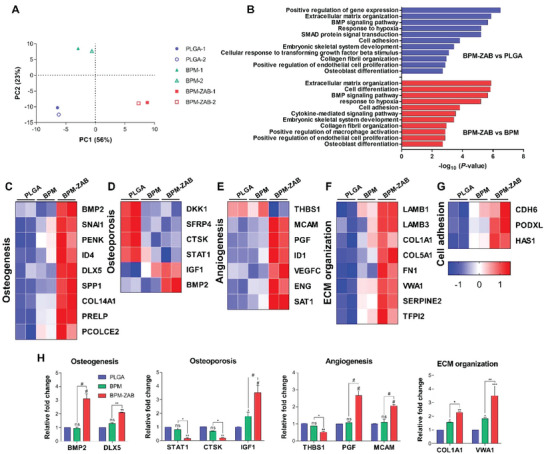
The molecular mechanism of the BPM‐ZAB using hBMSCs. A) Principal Component Analysis (PCA) plot for variation between each scaffold. The distance is proportional to the difference between gene expression profiles. B) Gene Ontology (GO) enrichment analysis of the relevant upregulated genes in the scaffolds with hBMSCs; BPM‐ZAB versus PLGA (blue) and BPM‐ZAB versus BPM (red). C–G) Heatmap of the classified genes according to the biological signal: low (blue) and high (red) expression. Data are presented with two samples per group (*n* = 2). H) Quantitative real‐time PCR (RT‐qPCR) analysis of classified genes according to their function. #*p* < 0.0001, ***p < 0.001, **p < 0.01, and *p < 0.05 indicate statistically significant differences (*n* > 3), respectively.

To verify the potential mechanisms of the BPM‐ZAB in vitro, we examined gene expression changes in hBMSCs cultured on each scaffold for 3 days via whole transcriptome RNA‐sequencing. The Principal Component Analysis (PCA) presented that the distinct gene expression was correlated with the types of scaffolds, implying that the composition of the scaffold and the immobilization of ZAB could affect the regulation of various gene expressions (**Figure** **4**A). The total gene heatmap showed the differentially expressed genes based on clustering with 41 upregulated and 33 downregulated genes on PLGA versus BPM‐ZAB (Figure S13, Supporting Information; log_2_foldchange > 1, < −1; *p*‐value < 0.05). The BPM‐ZAB exhibited the most distinguished gene expression with catalyzed NO releasing compared to PLGA and BPM. Moreover, Gene Ontology (GO) analysis was executed to investigate which biological functions were associated with the differential genes (Figure 4B). The GO analysis included extracellular matrix organization, response to hypoxia, cell adhesion, positive regulation of endothelial cell proliferation and, BMP signaling pathway, collagen fibril organization, and osteoblast differentiation. On the basis of preceding results, the scaffolds could regulate a various cellular signaling pathways. The heatmap of specific function‐related genes exhibited that ZAB upregulated osteogenesis, angiogenesis, ECM organization, cell–cell adhesion, and downregulated osteoporosis compared to PLGA and BPM (Figure 4C–G). The relative expression of functionalization‐related genes was determined using RT‐qPCR (Figure 4H). In particular, the osteoporotic markers, STAT1 and CTSK, were notably inhibited on the BPM‐ZAB. Taken together, BPM‐ZAB could enhance expressions of positive cellular signals and suppressed expressions of osteoporotic signals in comparison with BPM and PLGA.

### 2.4 In Vitro Osteogenic Differentiation, Antibacterial Effects, and Inhibition of Osteoclast Formation

**Figure 5:**
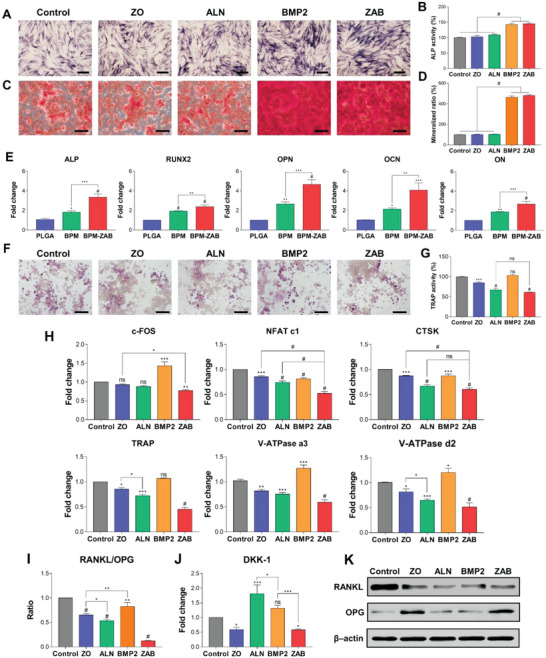
Osteogenic differentiation of the BPM‐ZAB using hBMSCs in vitro. A,B) The ALP staining and its quantification were conducted with ZO, ALN, BMP2, and ZAB for 7 days of osteogenic differentiation of hBMSCs. C, D) Alizarin Red S staining and its quantification performed with ZO, ALN, BMP2, and ZAB for 21 days of osteogenic differentiation of hBMSCs. E) The mRNA expression levels related to osteogenesis quantified by RT‐qPCR in 21 days of osteogenic differentiation; alkaline phosphatase (ALP), runt‐related transcription factor 2 (RUNX2), osteocalcin (OCN), osteopontin (OPN), and osteonectin (ON). Tartrate‐resistant acid phosphatase (TRAP) staining for detection of osteoclast activity (*n* = 3): F) TRAP staining images and G) quantified data treated with M‐CSF and RANKL cultured for 7 days. H) Osteoclast differentiation‐related gene expressions by RT‐qPCR after 7 days of osteoclastogenesis induction (*n* = 3); Fos proto‐oncogene (c‐FOS), nuclear factor of activated T cells 1 (NFAT c1), cathepsin K (CTSK), TRAP, V‐ATPase subunit a3, and d2. I) Inhibition mechanism of excessive osteoclast formation (*n* = 3): quantified RANKL/OPG expression ratio. J) Gene expression of DKK‐1 by RT‐qPCR. K) Western blot analysis of RANKL and OPG expression levels. #*p* < 0.0001, ***p < 0.001, **p < 0.01, and *p < 0.05 indicate statistically significant differences (*n* > 3), respectively.

The osteogenic capacity was analyzed using the ALP activity and mineralization with hBMSCs (**Figure** **5**A–D) to assess the osteogenic effects of ZAB and each scaffold. The BMP2 and ZAB were strongly stained compared to groups without BMP2. The ALP activity increased about 1.5‐fold with BMP2 and ZAB than in control (Figure 5B). The BMP2 and ZAB showed a better mineralization capacity, indicating that the BMP2 is the main factor of osteogenic differentiation (Figure 5C,D).

In the qPCR analysis on the 3D scaffold, the BPM‐ZAB exhibited notably upregulated gene expression levels of osteogenic markers, including ALP, RUNX2, OCN, OPN, and ON. The mRNA expression levels of BPM‐ZAB after 7 days (Figure S14, Supporting Information) were 2.17‐, 2.37‐, 3.60‐, 3.42‐, and 3.68‐fold higher than that of the PLGA, respectively. On day 21 (Figure 5E), the expression levels were upregulated by 3.35‐, 2.39‐, 4.07‐, 4.66‐, and 2.68‐fold in BPM‐ZAB than the PLGA, respectively.

The inorganic nanoparticles in the BPM‐ZAB imparted bactericidal activity, enhancing bone regeneration by reducing infection. Since the mMH and ZAB supplied Mg^2+^ and Zn^2+^ ions, well‐known as bactericidal metal ions, the BPM‐ZAB demonstrated antimicrobial activity against *S. saprophyticus* and *E. coli* (Figure S15, Supporting Information).

Tartrate‐resistant acid phosphatase (TRAP) is a marker expressed throughout osteoclast differentiation.^[^ ^19^ ^]^ TRAP staining was performed on macrophages differentiated with macrophage colony‐stimulating factor (M‐CSF) and RANKL (Figure 5F,G). The multinucleated cells were observed in the control and BMP2, and the ALN‐loaded groups (ALN and ZAB) remarkably obstructed the differentiation of osteoclasts.

The expression levels of CTSK, NFATc1, c‐FOS, and V‐ATPase a3 and d2 were quantified (Figure 5H) using the same condition. ZO and ALN could inhibit the expression of osteoclast‐related genes, but BMP2 slightly upregulated c‐FOS, TRAP, and V‐ATPase subunits compared to the control. These results highlight that the ZAB could considerably suppress the differentiation of the macrophages into osteoclasts via a combination of active molecules. In addition, ALN, BMP2, and ZAB notably downregulated the expression of RANKL. The expression of OPG increased by about 1.29‐ and 1.37‐fold in ZO and ZAB (Figure 5I and Figure S16, Supporting Information). To identify the mechanism of OPG/RANKL expression change, the Wnt/*β*‐catenin pathway‐related genes were assessed (Figure 5J and Figure S17, Supporting Information). Among the genes, DKK‐1, an inhibitor of the Wnt signaling pathway, was downregulated by ZO and ZAB. Consequently, these RANKL/OPG expression trend was also observed at the protein level (Figure 5K).

### 2.5 In Vivo Vascularization and Anti‐Inflammatory Activity of BPM‐ZAB on Normal Rats and Rats with Osteoporosis

**Figure 6:**
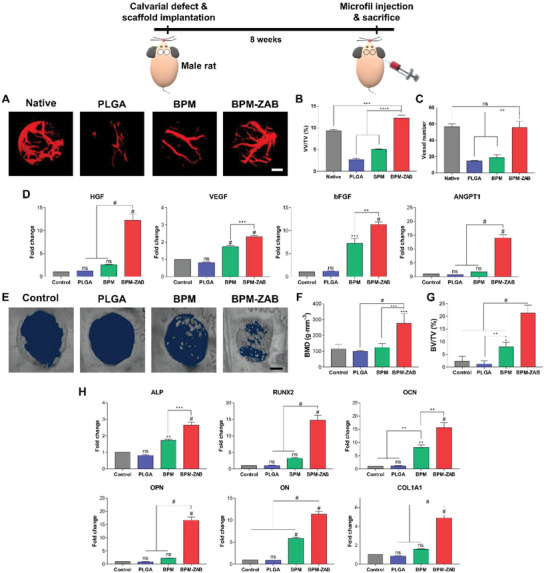
In vivo vascularization and anti‐inflammation of BPM‐ZAB on normal rats and rats with osteoporosis in normal rat (Model 1). A) 3D micro‐computed tomography (micro‐CT) reconstruction images of vascular (red) on normal rat calvarial defect at week 8 after implantation (scale bar, 1000 µm). B) Quantification of vessel density (VV/TV; vessel volume (VV), total volume (TV) (*n* = 3)). C) Quantification of vessel number (*n* = 3). D) Gene expression related to angiogenesis on the normal rat at week 8 (*n* = 3); HGF, VEGF, basic fibroblast growth factor (bFGF), and ANGPT1. E) Representative micro‐CT images of rat calvarial defects showing mineralized new bone at week 8 post‐implantation (scale bar, 1000 µm). F) Quantification of bone mineral density (BMD). G) Quantification of bone volume density (bone volume (BV)/total volume (TV)) (*n* = 3). H) Gene expression related to osteogenesis on the normal rat at week 8: ALP, RUNX2, OCN, OPN, ON, and COL1A1 (*n* = 3). #*p* < 0.0001, ***p < 0.001, **p < 0.01, and *p < 0.05 indicate statistically significant differences, respectively.

**Figure 7:**
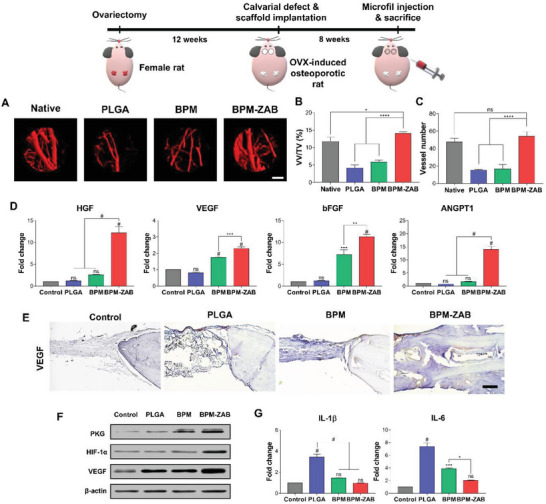
In vivo vascularization and inflammation effects of the BPM‐ZAB in osteoporotic rat (Model 2). A) 3D micro‐computed tomography (micro‐CT) reconstruction images of vascular (red) on an ovariectomy‐induced rat with osteoporosis and calvarial defect 8 weeks after implantation (scale bar, 1000 µm). B) Quantification of vessel density (VV/TV) (*n* = 3). C) Quantification of vessel number (*n* = 3). D) Gene expression related to angiogenesis on a rat with osteoporosis at week 8; HGF, VEGF, bFGF, and ANGPT1 (*n* = 3). E) Immunohistochemistry (IHC) staining of VEGF in rat calvarial defect (scale bar, 100 µm). F) Western blot analysis for markers related to nitric oxide (PKG, HIF‐1*α*, and VEGF). G) Gene expression related to inflammation on OVX rat at week 8 [Interleukin 1 beta (IL‐1*β*) and interleukin 6 (IL‐6)] (*n* = 3). #*p* < 0.0001, ***p < 0.001, **p < 0.01, and *p < 0.05 indicate statistically significant differences, respectively.

**Figure 8:**
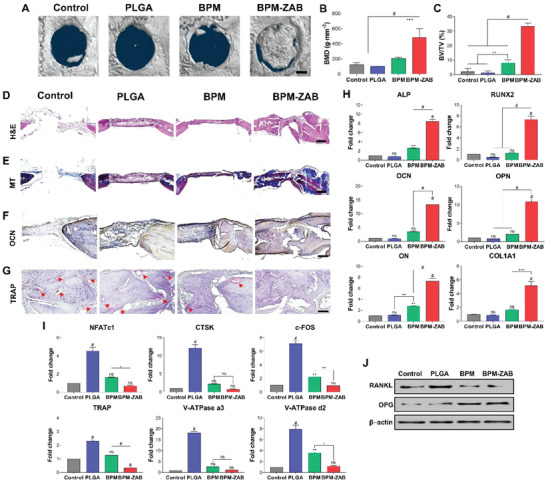
In vivo new bone formation ability of the BPM‐ZAB in osteoporotic rat Model 2. A) Representative micro‐CT images of ovariectomy‐induced rats with osteoporosis and calvarial defects showing mineralized new bone at 8 weeks post‐implantation (scale bar, 1000 µm). B) Quantification of BMD (*n* = 4). C) Bone volume density (BV/TV) quantification (*n* = 4). Histological analysis with D) Hematoxylin and Eosin (H&E), E) Masson's Trichrome (MT), F) osteocalcin (OCN; IHC), and G) tartrate resistant acid phosphatase (TRAP, red arrow). H) Gene expression related to osteogenesis in rats with osteoporosis at week 8 after implantation: ALP, RUNX2, OCN, OPN, ON, and COL1A1 (*n* = 3). I) Gene expressions related to osteoclast activation: NFATc1, CTSK, c‐FOS, TRAP, and V‐ATPase a3 and d2 (*n* = 3). J) The protein expression of RANKL and OPG was determined using Western blotting. #*p* < 0.0001, ***p < 0.001, **p < 0.01, and *p < 0.05 indicate statistically significant differences, respectively.

We implanted three types of scaffolds with hBMSCs on a 4 mm defect in the calvaria of the normal (**Figure** **6**) rat and rat with OP (**Figures** **7** and **8**). After 8 weeks of scaffold implantation, the contrast medium was injected to detect the vessels (Figures 6A and 7A). The 3D micro‐CT reconstruction images showed newly formed vessels (red) at the defect site. In normal rats and rats with OP, the BPM‐ZAB displayed a larger vessel volume/tissue volume (VV/TV) than in the PLGA, BPM, and native (without any damage) groups (Figures 6B and 7B). The vessel numbers were higher than in the PLGA and BPM groups, and there was an insignificant difference with the native group, which suggested that ALN indirectly supported vascularization in the osteoporotic environment through the inhibition of osteoclast activation, and that ZO directly induces vascular regeneration through NO released from endogenous donors (Figures 6C and 7C).

RT‐qPCR was performed to detect mRNA expression levels of angiogenic markers, including VEGF, HGF, ANGPT1, and bFGF (Figures 6D and 7D). In the PLGA and BPM groups of the osteoporotic model, no significant difference was presented in comparison with the control. In contrast, the relative mRNA expression of all markers remarkably increased in the BPM‐ZAB. Immunohistochemistry (IHC) staining revealed that VEGF production in vivo was upregulated by the BPM‐ZAB (Figure 7E). The protein markers related to the NO‐cGMP signaling pathway (PKG, HIF‐1*α*, and VEGF) were also upregulated on the BPM‐ZAB (Figure 7F).

Inflammation is the first response induced after damage or infection and is an essential process for protecting the body.^[^ ^20^ ^]^ The proinflammatory cytokine‐induced inflammation has been shown to have a crucial impact on the regeneration of various tissues.^[^ ^21^ ^]^ However, excessive inflammation could reduce regenerative capacity and rate.^[^ ^21b^ ^]^ In the osteoporotic model, the gene expression of IL‐1*β* and IL‐6 was higher by about 1.29‐ and 1.37‐fold in PLGA (Figure 7G). In contrast, the BPM and BPM‐ZAB downregulated the expression of proinflammatory cytokines because mMH contributed to regeneration by preventing tissue acidification by degradation byproducts of PLGA. In summary, in vivo vascularization and anti‐inflammatory activity of the BPM‐ZAB were much higher in rats with OP than in normal rats.

### 2.6 In Vivo Bone Regeneration Effect of BPM‐ZAB on Normal Rats and Rats with Osteoporosis

We investigated new bone formation at the defect site by micro‐CT at 8 weeks of implantation (Figures 6E and 8A). The PLGA group exhibited lower regenerated bone than the control group. The BPM‐ZAB group showed an outstanding regeneration compared to all other groups. In both normal and osteoporotic models, the bone mineral density (BMD) and bone volume/tissue volume (BV/TV) of the BPM‐ZAB were remarkably higher (276.93 g cm^−1^, 21.325%; 483.78 g cm^−1^, 33.31%, respectively) than other groups (Figures 6F–G and 8B,C). In particular, the new bone was formed better in the osteoporotic rat model than the normal one in the BPM‐ZAB group. The radiographic analysis was supported by histological analysis of newly formed bone tissue with H&E, MT, and IHC (Figure 8D–G). The scaffolds were almost mineralized with new bone at 8 weeks in the BPM‐ZAB group. To further investigate the osteogenic differentiation‐related gene expression, qPCR was performed with ALP, RUNX2, OCN, OPN, ON, and COL1A1 (Figures 6H and 8H). PLGA showed negligible difference compared to the control, and the BPM‐ZAB displayed 2.66‐, 14.74‐, 15.68‐, 16.66‐, 11.35‐, and 4.88‐fold higher expression in the osteoporotic model (Figure 8H).

The relative mRNA expressions of osteoclast‐related genes were also quantified (Figure 8I). In the PLGA, all genes were notably upregulated, and the BPM exhibited a slight increase compared with the control. Like in vitro analysis, protein expression of OPG and RANKL was upregulated and downregulated in the BPM‐ZAB, respectively (Figure 8J).

## 3 Discussion

In general, there are several studies on normal bone regeneration using the scaffolds; however, there are relatively few studies on osteoporotic conditions. Although amino BPs are typically employed as first‐line therapy for the treatment of osteoporosis, but when BPs are used bone regeneration may be disrupted.^[^ ^22^ ^]^ While these are effective in increasing bone density, it has been reported they can also increase the risk of fracture due to excessive bone density over time, in addition to having side effects such as joint pain and hypocalcemia when taken for a long time. A novel bioinspired scaffold with bioactive agents was proposed to significantly improve bone regeneration even in osteoporotic bone, thus resolving this challenge.

Stem cell therapy is an attractive way to regenerate various tissues, but it is restricted owing to the short persistency of the cells on the scaffolds, especially in OP.^[^ ^23^ ^]^ In our previous study, the pH of the PLGA (lactide:glycolide, 50:50) scaffold decreased to 3.5, and the scaffold was fully degraded by day 60.^[^ ^24^ ^]^ In an aqueous solution, the ester bonds of PLGA are degraded by hydrolysis. Since the side methyl groups on the polymer chain add hydrophobicity, more lactide composition in PLGA can absorb less water, resulting in slower degradation.^[^ ^25^ ^]^ Consequently, BPM‐ZAB exhibited a longer duration than the previous PLGA‐based scaffold due to a higher lactide/glycolide ratio (75:25) and molecular weight, resulting in a prolonged retention time to 10 weeks. PLGA exhibited a significantly faster degradation under dynamic condition than under static conditions.^[^ ^26^ ^]^ Therefore, the 10 weeks degradation period of the in vitro experiment would be appropriate for the 8 weeks in vivo bone regeneration experiment.

In this study, we opted for ALN as an anti‐osteoporotic agent to facilitate local bone regeneration in ovariectomized rats. The amino‐bisphosphonates, such as risedronate, zoledronate, and ALN, have been reported to cause osteonecrosis of the jaw (ONJ) in 2003. Because the anti‐angiogenic agents were added to the ONJ‐inducing medications list, the medication‐related osteonecrosis of the jaw (MRONJ) has been newly defined by the American Association of Oral and Maxillofacial Surgeons.^[^ ^27^ ^]^ We hypothesized that the anti‐angiogenic effect is a major cause of MRONJ; therefore, we designed a sustained NO release system via ZO nanoparticles to induce vascularization. Yang et al. described that the ZO could induce the most active NO production among the metals and metal oxides.^[^ ^28^ ^]^ NO exhibits a protective or noxious influence on tissue in a concentration‐dependent manner. In mammalian cells, low concentrations of NO (1–30 nm) can induce angiogenesis, cell survival, and proliferation through the activation of soluble guanylyl cyclase (sGC) and increase cGMP generation whereas high concentration (µm) can induce apoptosis or cell cycle arrest.^[^ ^29^ ^]^ We showed that the NO‐cGMP signaling pathway and protecting degradation of HIF‐1*α* are major mechanisms of inducing angiogenesis with sustained release of NO (Figure 3H–J). Continuous NO production induces activation of sGC leading to the cGMP signaling pathway.^[^ ^30^ ^]^ The signaling molecules like cGMP and PKG are upregulated, resulting in high expression of angiogenic factors, VEGF and HIF‐1*α*. In addition to this role, NO directly disrupts *S*‐nitrosylation of HIF‐1*α*, signifying that NO reacts with the residual cysteine group of HIF‐1*α*. As a result, it suppresses degradation by inhibiting the ubiquitination of HIF‐1*α*. NO‐cGMP and HIF‐1*α* pathways suggest that NO is a crucial signaling molecule in angiogenesis.

In terms of bone homeostasis, excessive RANKL activates the formation of osteoclasts and hinders the differentiation of hBMSCs into osteoblasts.^[^ ^31^ ^]^ The interaction between RANKL and its receptor promotes the formation of activated osteoclasts, guiding bone resorption and loss. The OPG has the opposite ability to RANKL, including inhibition of osteoclast differentiation and accelerating osteoclast apoptosis.^[^ ^32^ ^]^ In the osteoblast lineage, it is reported that Wnt/*β*‐catenin signaling promotes osteogenesis by upregulating OPG and downregulating RANKL.^[^ ^33^ ^]^ We demonstrated that ZAB not only inhibits osteoclast by inducing apoptosis with ALN but also increases the expression of OPG by upregulating the expression of DKK‐1 with ZO (Figure 5I–K). This was also observed in vivo (Figure 8J) and directly impacted anti‐osteoclastogenesis and osteogenesis for bone regeneration in the osteoporosis model.

The multifunctional biomaterials are necessary for the successful regeneration of osteoporotic bone.^[^ ^34^ ^]^ In the present study, we prepared the bioinspired PLGA/MH/ECM scaffold immobilized with ZO/ALN/BMP2 nanoparticles. The PLGA was combined with organic/inorganic extracellular matrices (bECM and DBM in the ratio of 7:3) to enhance the osteogenic differentiation of hBMSCs and the mechanical properties of the scaffolds. The mMH could effectively neutralize the acidification caused by the PLGA degradation products. The ZAB has outstanding angiogenic, osteoinductive, and anti‐osteoclastogenic effects. Therefore, the combination of BPM and ZAB showed a noticeable synergistic effect on osteogenesis in vitro (Figure 5A–F) and in vivo (Figures 6 and 8). Interestingly, in normal and osteoporotic rat calvarial defect models, since we designed the ZAB for osteoporotic bone regeneration using ALN, the new bone was formed better in the OVX‐induced osteoporotic model than in the normal model (Figures 6, 7, 8). Because the ZAB can regulate bone homeostasis through inhibition of osteoclastogenesis, the bone was effectively regenerated in osteoporotic fracture.

## 4 Conclusion

We have successfully prepared a novel nitric oxide‐releasing bioinspired scaffold with regulation of homeostasis to achieve effective bone regeneration in the osteoporotic rat femoral defect model. The main goal of implantable biomaterial design is to prepare an ideal scaffold that is easily applicable and has various effects. Consequently, we achieved our aim by showing the potential of BPM‐ZAB to be applied for osteoporotic bone defects through antibacterial effect, NO production ability, osteoclast inhibition capacity, and osteogenic effects. Taken together, this bioactive agents‐laden bioinspired scaffold is preferable for bone regeneration exquisitely compared to the previous approach. It can potentially improve stem cell therapy, especially in patients with osteoporosis.

An advanced strategy for the preparation of bone graft material using biomimetic scaffolds was proposed. A functional nanoparticle that consists of three bioactive molecules demonstrated outstanding angiogenic and osteogenic effects. The nanoparticle‐immobilized bioinspired scaffold exhibited multifunctional activity in anti‐inflammation, angiogenesis, anti‐osteoclastogenesis, and bone regeneration.

## 5 Experimental Section

### Materials

Poly(D,L‐lactic‐co‐glycolic acid) (lactide:glycolide = 75:25, I.V. = 0.8–1.2) was purchased from Evonik Ind. (Essen, Germany). MH, L‐ascorbic acid, dexamethasone, ZO nanopowder (677450), LB Broth (L3022), LB Broth with Agar (L2897), Nutrient broth (70122), and Nutrient Agar (70148) were purchased from Sigma‐Aldrich (MO, USA). ALN(13642) was purchased from Cayman Chemical Co. (MI, USA). RA was purchased from TCI Product (Tokyo, Japan). BMP2 (Mw = 26 kDa) was supplied by CG BIO (Seongnam, Korea). bECM was supplied by Oscotec Inc. (Seongnam, Korea). DBM was supplied by Hans Biomed Co. (Seoul, Korea). D‐Plus cell counting kit 8 (CCK‐8) cell viability assay kit was obtained from Dongin LS (Seoul, Korea). The TRACP & ALP double‐stain kit (MK300) and TRACP & ALP activity kit (MK301) were obtained from Takara Korea Biomedical Inc. (Seoul, Korea).

### Preparation of the ZO/ALN/BMP2 Nanoparticles

The ZAB were synthesized using the ionic interaction and EDC/Sulfo‐NHS reaction. ZO was pulverized using an Ultra‐high nano‐disperser (Laboratory Agitator Mill MiniCer, NETZSCH, Germany). Then, 3 mg of ZO was mixed with 15 mg of sodium citrate tribasic dihydrate (S4641, Sigma‐Aldrich) in 5 mL of 100 mm MES buffer (pH 6.5), and the EDC (5 mm) and Sulfo‐NHS (15 mm) were added. After that, 1 mg of ALN was dissolved with the particles in 5 mL of 10 mm NaCl solution. After 24 h, unreacted molecules were removed using 100 kDa Amicon Ultra 15 centrifugal filter units (Merck, Germany). Afterward, 3 mg of ZO modified with citrate and ALN was reacted by ionic interaction with 10 µg of BMP2 in 10 mm NaCl solution. The ZAB was then lyophilized for further use. The obtained 1 mg of ZAB contained about 386 µg of ZO, 611 µg of ALN, and 2.83 µg of BMP2.

### Characterization of ZO/ALN/BMP2 Nanoparticles

The hydrodynamic size and zeta potential of ZAB were determined using Zetasizer Nano ZS (Malvern Instruments Ltd., Worcestershire, UK). The morphology of ZAB was observed using FE‐SEM (Carl Zeiss, Germany) and HR‐TEM (Tecnai, FEI Company, OR, USA) equipped with a TEM‐EDS analysis system. The structure of the ZAB was characterized by PXRD (D2 Phaser, Bruker, MA, USA). The ZAB was characterized using Fourier transform‐infrared spectroscopy (FTIR) (Spectrum two, PerkinElmer, UK).

### Preparation of Biomimetic PLGA/MH/ECM Scaffold

mMH was synthesized following the process mentioned in the previous study using RA.^[^ ^35^ ^]^ The scaffolds were prepared by the freeze‐drying method. The ice particles (200–300 µm), porogen for porous scaffold, were prepared by spraying deionized water into liquid nitrogen. Then, 0.5 g of PLGA was dissolved in a 0.3 m dichloromethane (DCM) solution. In addition, 20 wt% of mMH, 50 wt% of bECM, and 21 wt% of DBM were mixed with ice particles in 0.3 m DCM solution with PLGA. The PTFE molds (Φ5 × 2 mm^2^) were filled with the mixture. The molds were lyophilized for 2 days; then, the porous scaffolds were obtained.

### Preparation of ZAB‐Immobilized Bioinspired Scaffold

To immobilize ZAB on biomimetic PLGA/mMH/bECM scaffold, the scaffolds were hydrated by 70% ethanol and deionized water in order. Then, 500 µg of ZAB and BPM scaffold were immersed together in a supersaturated calcium phosphate solution [NaCl (8.065 g), CaCl_2_ (0.554 g), and Na_2_HPO_4_ (0.284 g), buffered with Tris (50 mm; pH 7.4)]. After immobilization, the bioinspired scaffolds were washed with distilled water and then freeze dried for 1 day.

### Characterization of Biomimetic PLGA/MH/ECM Scaffold

The surface morphology and calcium deposition of the scaffolds were observed by FE‐SEM. The mechanical property of the scaffolds was analyzed by a universal testing machine (UTM; Instron‐4464, MA, USA) with 1 N load cell. The porosity of the scaffolds was measured using a mercury intrusion porosimeter (AutoPore IV 9520, Micromeritics, GA, USA). The thermal property of the scaffolds was estimated using a thermogravimetric analyzer (TGA, TGA 4000, Perkin Elmer, MA, USA). Mass and pH changes were measured in 500 µL of PBS solution (pH 7.4) for 70 days to evaluate neutralizing ability of the scaffolds. The elemental mapping was executed using FE‐SEM (S‐4800, Hitachi, Japan) equipped with energy dispersive spectroscopy (EDS). The elemental compositions of scaffolds such as zinc, magnesium, calcium, and phosphorous were measured by inductively coupled plasma‐optical emission spectroscopy (ICP‐OES, Optima 8000, Perkin Elmer, MA, USA). The release of the BMP2 was determined using an ELISA (RHF913CKX, Antigenix America, NY, USA). The release of the ALN and ZO was determined using ICP‐OES.

### Characterization of Nitric Oxide Release

To visualize the NO generating ability of the 3D scaffold, the fluorescence imaging system (FOBI, CellgenTEK Co., Daejeon, Korea) was used. Then, 50 µm of *S*‐nitroso‐*n*‐acetylpenicillamine (SNAP) and L‐glutathione reduced (GSH) were mixed. The NO‐sensitive fluorescent dye (DAF‐FM, 18767, Cayman Chemical, MI, USA) was mixed with a concentration of 50 µm. Each sample was incubated in a 96‐well plate with the above‐mixed solution for 6 h. After the reaction, the plate was visualized by FOBI at the blue channel. A Sievers NOA 280i Chemiluminescence Nitric Oxide Analyzer (GE Analytical Instrument, Boulder, CO, USA) was used to quantify the NO release by a chemiluminescence reaction between NO and ozone. Before analysis, the instrument was calibrated using a two‐point linear calibration with a NO zero filter (0 ppm NO) as the blank value and 45 ppm of NO standard gas (nitrogen balance) as the known value. ZAB was immersed in the NOA sample flask containing deoxygenated 0.01 m PBS solution (pH 7.4) at 37 °C with 10 µm GSH and SNAP.

### Effect of Nitric Oxide on HUVECs

The cells were seeded on a 96‐well plate to confirm the effects of the NO on HUVECs. The 10 µm of GSH and SNAP was treated with ZAB and its components. The CCK‐8 assay was conducted at a predetermined time. The angiogenic effect of NO was confirmed with tube forming assay, qPCR, and ELISA. The cells were seeded on a 24‐well plate, and then the NO donor and ZAB were treated. After 48 h, the medium was filtered using a 0.22 µm syringe filter. The expression of ANGPT1, VEGF, and HGF was evaluated using ELISA.

### Osteogenic Differentiation: ALP Staining and Activity

To assess the early osteogenic ability of hBMSCs and the effect of ZAB, ALP staining was conducted on day 7 after osteogenic differentiation in an osteogenic medium (OM; DMEM/Low glucose supplemented with 10% FBS, 1% 520 A/A, 50 µm L‐ascorbic acid, 0.1 µm Dexamethasone, and 10 mm *β*‐glycerophosphate). The hBMSCs (5 × 10^4^ cells per well) were seeded on a 24‐well plate. After 1 day, the medium was replaced with OM. On day 7, the cells were washed with PBS solution, fixed with 10% formalin for 20 min, and rinsed with deionized water. The fixed sample was stained with a Takara ALP stain kit. The images of stained samples were captured with optical microscopy. For the measurement of ALP staining, the cells were lysed using a Takara ALP assay kit with the same condition and time point. The whole process followed the provided protocol.

### Osteogenic Differentiation on Mineralization: Alizarin Red S Staining and Quantification

To confirm the osteogenic ability of hBMSCs and the effect of ZAB, alizarin red S (ARS) staining was executed 21 days after osteogenic differentiation in an OM. With the same condition as ALP staining, the cells were fixed with 10% formalin for 20 min and rinsed with deionized water. The plate was incubated with 2% ARS solution for 20 min. The stained cells were observed using optical microscopy. To quantify the mineralization, the 10% cetyl pyridinium chloride (CPC; C0732, Sigma‐Aldrich) was added to each well and incubated for 15 min. The colorimetric measurement was read at a wavelength of 562 nm using a microplate reader (Spectramax M2, Molecular Devices, CA, USA).

### RNA Extraction for RT‐qPCR

The RNA from 2D‐cultured cells and 3D scaffolds was extracted using a Universal RNA Extraction kit (K‐3141, Bioneer, Korea) and Trizol reagent (15596018, Thermo Fisher Scientific, MA, USA). The concentration of RNA was determined by spectrophotometer (ND‐1000; Thermo Fisher Scientific, MA, USA). Then, 100 ng of RNA from each sample was reversely transcribed to cDNA using a PrimeScript RT Reagent kit (Perfect Real‐Time). The RT‐qPCR was performed using Power SYBR Green PCR Master Mix 542 (Applied Biosystems, CA, USA) with a QuantStudio 3 real‐time PCR instrument (Applied 543 Biosystems, CA, USA). The relative gene expressions were calculated with 18S rRNA as a reference gene. All primer sequences are described in Table S2 (Supporting Information).

### Antibacterial Effect

Gram‐positive bacteria, *S. saprophyticus*, and Gram‐negative bacteria, *E. coli*, were obtained from Korean Collection for Type Cultures (KCTC). *E. coli* was incubated in LB Broth (Lennox) at 37 °C with aeration. *S. saprophyticus* was incubated in nutrient broth at 37 °C with aeration. The antibacterial ability test was conducted following the previously reported method.^[38]^

### Inhibiting Effect on Osteoclastogenesis

The osteoclastogenesis was induced using 70 ng mL^−1^ of receptor activator of nuclear factor‐*κ*B ligand (RANKL) and 50 ng mL^−1^ of macrophage colony‐stimulating factor (M‐CSF). To differentiate osteoclast precursor cells into activated osteoclast, the RAW264.7 cells, murine macrophage cell line, were obtained from Korean Cell Line Bank (KCLB, Seoul, Korea) and seeded into a 96‐well plate (3 × 10^3^ cells per well). After 1 day, the cells were treated with differentiation factors and each component of ZAB. After 5 days of treatment, the cells were fixed with 4% paraformaldehyde, rinsed with deionized water, and stained with a Takara TRAP stain kit. The stained samples were observed with optical microscopy. The cells were lysed using a Takara TRACP assay kit to quantify TRAP activity. The whole process was conducted following the provided protocol.

### Cells Seeding on the Scaffolds for In Vitro Assay and In Vivo Study

Fabricated scaffolds were immersed in 70% ethanol, DW, and PBS solution serially for hydration and sterilization. After soaking, the 5 × 10^5^ hBMSCs were seeded on each scaffold. After 1 h, medium was added in a 24 well plate. After 1 day, the scaffolds were used for rat calvarial bone defect.

### The Surgical Procedure of Normal Rat Calvarial Bone Defect (In Vivo Model 1)

The experimental protocol for the use of animals was approved by the Institutional Animal Care and Use Committee of CHA university (IACUC210099). The animals were anesthetized with isoflurane (Terrell Isoflurane, Piramal Critical Care Inc., PA, USA). The hair was shaved, and the exposed skin was sterilized with 70% ethanol. A midline sagittal incision was executed. The defects were drilled into 4 mm on both sides of rat calvaria at a certain distance from the sagittal suture line. The drilled calvarial disc was removed, and the scaffolds were implanted into the defect. The periosteum was closed with absorbable sutures, and the ad1epidermis of the implanted site was sutured with non‐absorbable sutures.

### Calvarial Defect in Rats with Osteoporosis (In Vivo Model 2)

Twelve weeks‐old female Sprague‐Dawley rats underwent ovariectomy (OVX). The experimental osteoporosis was induced by bilateral OVX under anesthesia. Three months after OVX, rats with the age of 24 weeks underwent the above surgical process for normal rat calvarial defect.

### Micro‐CT Scanning and Analysis

The obtained calvarial samples were fixed in 4% paraformaldehyde at 25 °C for 7 days for micro‐CT scanning. Images were obtained using Skyscan 1076 (Bruker, MA, USA). DataViewer and CTVox software (Bruker, MA, USA) were used to examine the structure of the sample for all X‐ray imaging. The BV/TV (%) and BMD (%) were measured with the CTAn.

### Histological Analysis

All fixed rat calvaria samples were decalcified with RapidCal immune (BBC Biochemical) solution with gentle shaking at 4 °C. The solution was changed every 2 days. After 21 days, the samples were dehydrated and embedded into paraffin. Next, 10 µm thick coronal sections were executed. The sectioned slides were stained with Hematoxylin & Eosin (Abcam, UK), Masson's Trichrome staining kit (VitroVivo Biotech), and immunohistochemistry staining (OCN, sc‐365797, Santacruz, TX, USA, 1:100). TRAP staining was performed with paraffin slides using TRAP staining kit (PMC‐AK04‐COS, Cosmo bio, Japan) following the provided protocol.

### Microfil Perfusion

The rats were anesthetized and perfused with Microfil (MV‐122; Flow Tech, MA, USA) to visualize the newly formed blood vessel at week 8. The ribs were opened using scissors, and the left ventricle was penetrated with a 21 G butterfly needle. A 50 mL of heparinized saline and 50 mL of 4% paraformaldehyde were completely perfused at a rate of 9 mL min^−1^ using a microfluidic syringe pump (NE‐1000‐ES; New Era Pump Systems Inc., NY, USA). Finally, 20 mL of Microfil was perfused at 2 mL min^−1^. The perfused samples were set overnight at 4 °C to cure the agent completely, and images were captured with micro‐CT scanning.

### Western Blot

All samples were lysed with RIPA buffer (9806S, cell signaling technology, MA, USA). Lysates were sonicated and centrifuged at 13 000 rpm for 10 min. Protein concentration was determined by a BCA assay. Membranes were blocked for 1 h on 5% skim milk in TBST buffer (Tris‐buffered saline with Tween‐20). Primary antibodies were as follows: mouse anti‐RANKL (ab239607, Abcam, 1:500 dilution), anti‐osteoprotegerin (Abcam, ab73400, 1:500 dilution), anti‐HIF‐1*α* (ab51608, Abcam, 1:500 dilution), and anti‐VEGF (sc‐7269, Santacruz, 1:250 dilution). Horseradish peroxidase‐conjugated anti‐mouse (7076S, cell signaling technology) and anti‐rabbit (7074P2, cell signaling technology) secondary antibodies were used with 1:5000 dilution.

### RNA‐Sequencing and Data Analysis

The RNA‐sequencing assay was performed by E‐Biogen Inc. (Seoul, Korea). Total RNA was isolated using Trizol reagent (Invitrogen, MA, USA). RNA quality was assessed using Agilent 2100 bioanalyzer (Agilent Technologies, Amstelveen, The Netherlands), and RNA quantification was performed using an ND‐2000 Spectrophotometer (Thermo Inc., DE, USA). The total 25737 of genes were analyzed. For each RNA sample, a library was constructed using a QuantSeq 30 mRNA‐Seq Library Prep kit (Lexogen, Vienna, Austria). Data mining and graphic visualization were performed using ExDEGA (E‐Biogen, Inc., Seoul, Korea).

### Statistical Analysis

All experiments were repeated at least three times independently. The results are shown as means ± SD. #*p* < 0.0001, ***p < 0.001, **p < 0.01, and *p < 0.05 indicate a statistically significant difference. Statistically significant differences were evaluated by one‐way ANOVA analysis of variance using the Tukey post hoc method in GraphPad Prism 7.0 software (GraphPad Software Inc., CA, USA).

### Data Availability 

The main data supporting the results of this study are available in the paper and its Supporting Information. The raw data are available from the corresponding authors on reasonable request.

## Conflict of Interest

The authors declare no conflict of interest.

## Supporting information

Supporting Information

## Data Availability

The data that support the findings of this study are available from the corresponding author upon reasonable request.
