# Supplementary material for: Nitric Oxide‐Releasing Bioinspired Scaffold for Exquisite Regeneration of Osteoporotic Bone via Regulation of Homeostasis
Source: Adv Sci (Weinh). 2022 Dec 29;10(6):2205336. doi: 10.1002/advs.202205336 (PMC9951336; doi:10.1002/advs.202205336)
Supplement: Supplementary file 1 — Supporting Information [file ADVS-10-2205336-s001.pdf]

Supporting Information

Nitric Oxide-Releasing Bioinspired Scaffold for Exquisite Regeneration of Osteoporotic Bone via Regulation of Homeostasis

*Jun-Kyu Lee†, Da-Seul Kim†, So-Yeon Park, Seung-Woon Baek, Ji-Won Jung, Tae-Hyung Kim, and Dong Keun Han**

Table S1. Quantification of the Mg, Ca, P, and Zn concentration in the scaffold with ICP-OES

	Mg (ppm)	Ca (ppm)	P (ppm)	Zn (ppm)
PLGA	-	-	-	-
BPM	167.91±1.55	352.02±3.08	143.57±8.80	-
BPM-ZAB	136.43±2.05	403.88±3.61	179.67±3.61	70.22±5.49

Table S2. List of primers used in the study for RT-qPCR.

Species	Gene	Primer	Sequence (5' - 3')
Human	VEGF	Forward	ACTGGACCCTGGCTTTACTG
		Reverse	TCTGCTCCCTTCTGTCGT
	HGF	Forward	CAGCATGTCCTCCTGCATC
		Reverse	TCTTTTCCTTTGTCCCTCTGC
	ANGPT1	Forward	TCCACATAGGAAATGAAAAGCA
		Reverse	CAGCACCGTGTAAGATCAGG
	ALP	Forward	ATCTCCTGACCCTCCCACTC
		Reverse	AGTGAGTGAGTGAGCAAGGC
	RUNX2	Forward	GCGCATTCCTCATCCCAGTA
		Reverse	GGCTCAGGTAGGAGGGGTAA
	OCN	Forward	GTGCAGCCTTTGTGTCCAAG
		Reverse	TCAGCCAACTCGTCACAGTC
	OPN	Forward	AGCAGAATCTCCTAGCCCCA
		Reverse	CTGGCTGTCCACATGGTCAT
	ON	Forward	CAAGAAGCCCTGCCTGATGA
		Reverse	TCTTCGGTTTCCTCTGCACC
	COL1A1	Forward	CAGGCTGGTGTGATGGGATT
		Reverse	CTCCATCTTTGCCAGCAGGA
Mouse	c-FOS	Forward	GGGACAGCCTTTCCTACTACC
		Reverse	AGATCTGCGCAAAAGTCCTG
	NFATc1	Forward	TCCAAAGTCATTTTCGTGGA
		Reverse	CTTTGCTTCCATCTCCCAGA
	CTSK	Forward	CCGAATAAATCTAGCACCCTTAGT
		Reverse	GAAACTTGAACACCCACATCC
	TRAP	Forward	CGTCTCTGCACAGATTGCAT
		Reverse	GGAACTTTGAAACGCAAACG
	V-ATPase a3	Forward	CCATATCCCTTTGGCATTGA
		Reverse	GAGAAAGCTCAGGTGGTTCG
	V-ATPase d2	Forward	AAGCCTTTGTTTGACGCTGT
		Reverse	TGAATGCCAGCACATTCATC
	RANKL	Forward	GAAGACACACTACCTGACTC
		Reverse	GGACAGAGTGACTTTATGGG
	OPG	Forward	AGCCCAGAAGAGATTGAGAG
		Reverse	CATGGTCTTCCTCAGACTGT
Rat	HGF	Forward	GATTGGATCAGGACCTTGTGA
		Reverse	CCATTCTCATTTTGTGTTGTTCA
	VEGF	Forward	ACTGGACCCTGGCTTTACTG
		Reverse	TCTGCTCCCCTTCTGTCGT
	bFGF	Forward	AAGAGCGACCCACACGTC
		Reverse	CCCTTGATGGACACAACTCC
	ANGPT1	Forward	ATGCGCCCTTATGCTAACAG
		Reverse	TTTAGATTGGAAGGGCCACA

	IL-1β	Forward	TCAAGCAGAGCACAGACCTG
		Reverse	CATGTCCTGGGGAAGGCATT
	IL-6	Forward	CCTGGAGTTTGTGAAGAACAACT
		Reverse	GGAAGTTGGGGTAGGAAGGA
	c-FOS	Forward	GGGACAGCCTTTCCTACTACC
		Reverse	GATCTGCGCAAAAGTCCTGT
	NFATc1	Forward	GAAGCCAGTACCAGCATTTCA
		Reverse	CGTCCGTGGGTTCTGTCT
	CTSK	Forward	CGACTATCGAAAGAAAGGCTATG
		Reverse	AAAGCCCAACAGGAACCAC
	TRAP	Forward	CTGGGTGGCTTCACATACGT
		Reverse	GTAAGCCTCTGTGGTTCCCC
	V-ATPase a3	Forward	CTTATCCCTTCGGCATTGAC
		Reverse	TTGAGGAAGCTCAGGTGGTT
	V-ATPase d2	Forward	GCAAAGCCAGCCTCCTAA
		Reverse	GTCAGTGGTCTGGAGATGAATTT

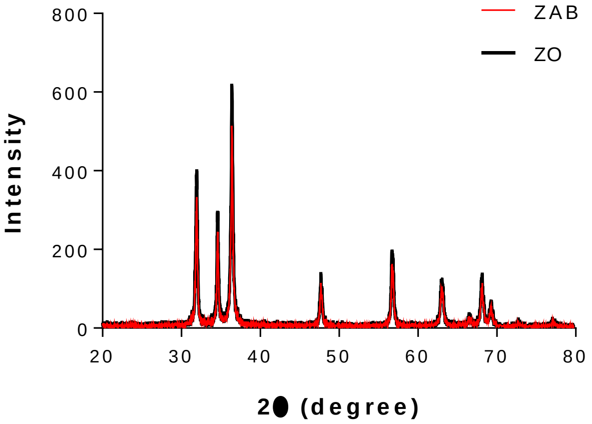

Figure S1. Powder X-ray diffraction (PXRD) pattern of ZO (black) and ZAB (red) nanoparticle.

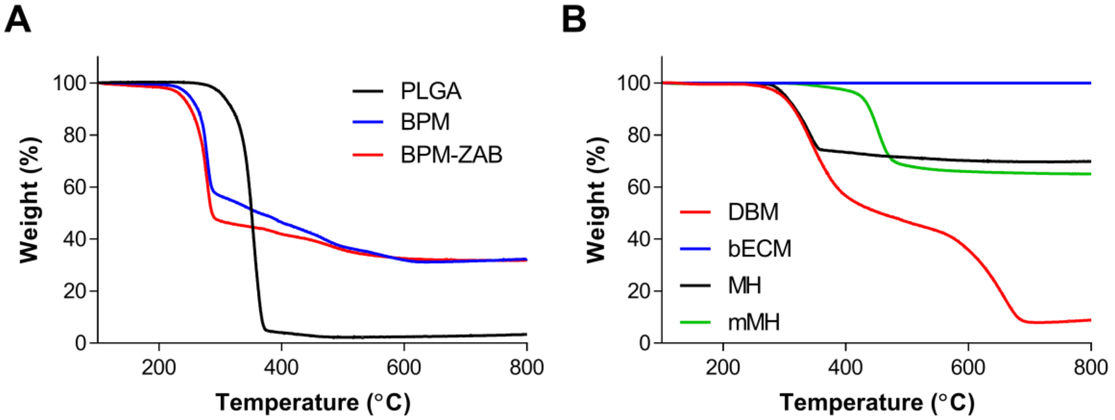

Figure S2. TGA plot of (A) the scaffolds and (B) ingredients of the scaffold.

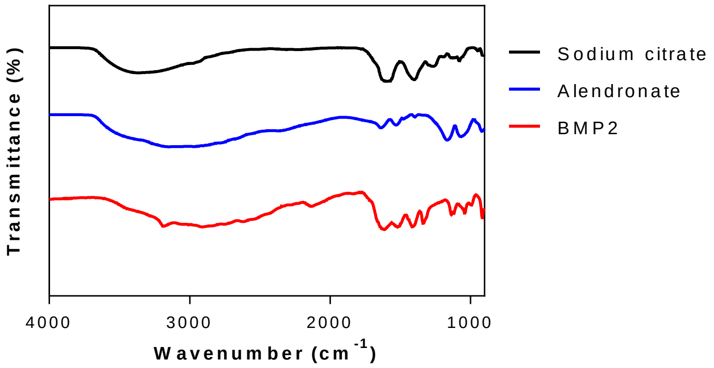

Figure S3. ATR-FTIR spectra of each materials. Sodium citric acid (CA, black), Alendronate (ALN, blue), Bone morphogenetic protein 2 (BMP2, red).

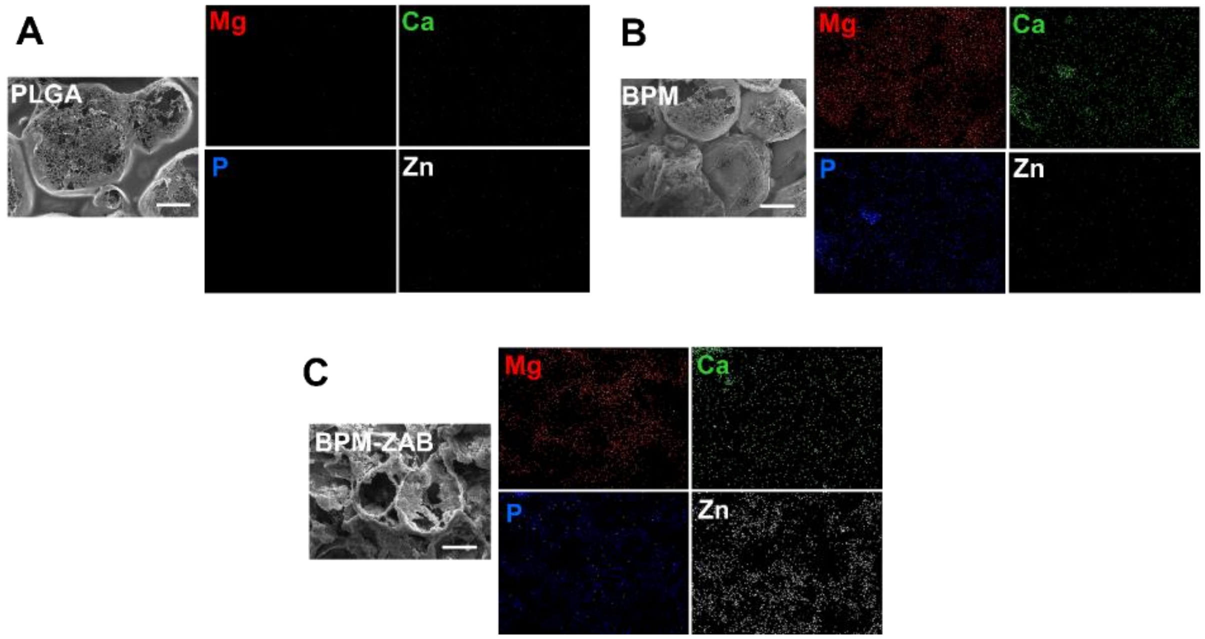

Figure S4. SEM-EDS elemental mapping images of the scaffolds. Magnesium (Mg, red), calcium (Ca, green), phosphorous (P, blue), zinc (Zn, white); scale bar = 100 μm.

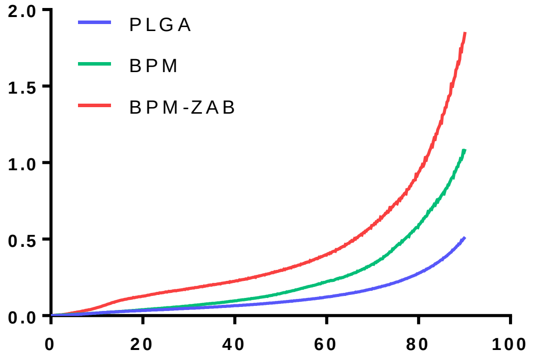

Figure S5. Representative stress-strain profiles for each scaffold.

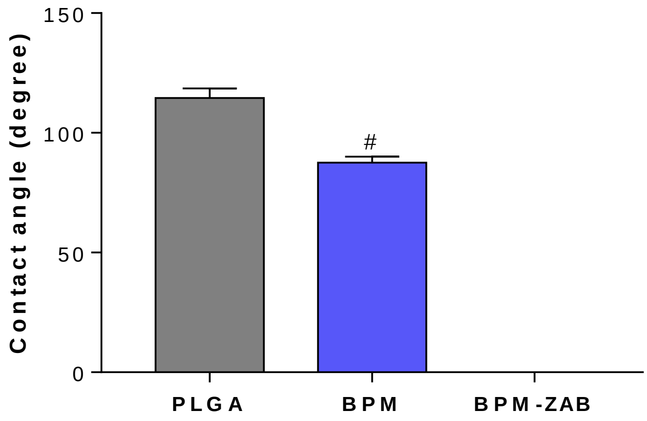

Figure S6. Water contact angle of the scaffolds (n=3). The graph shows the mean value of the contact angle measurements. #p < 0.0001, ***p < 0.001, **p < 0.01, and *p < 0.05 indicate statistically significant differences, respectively.

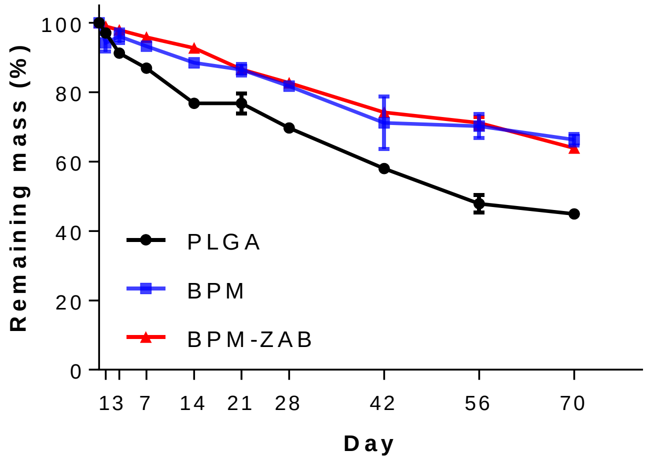

Figure S7. Changes in mass of the scaffolds during *in vitro* degradation process in PBS solution at 37 °C (n=3).

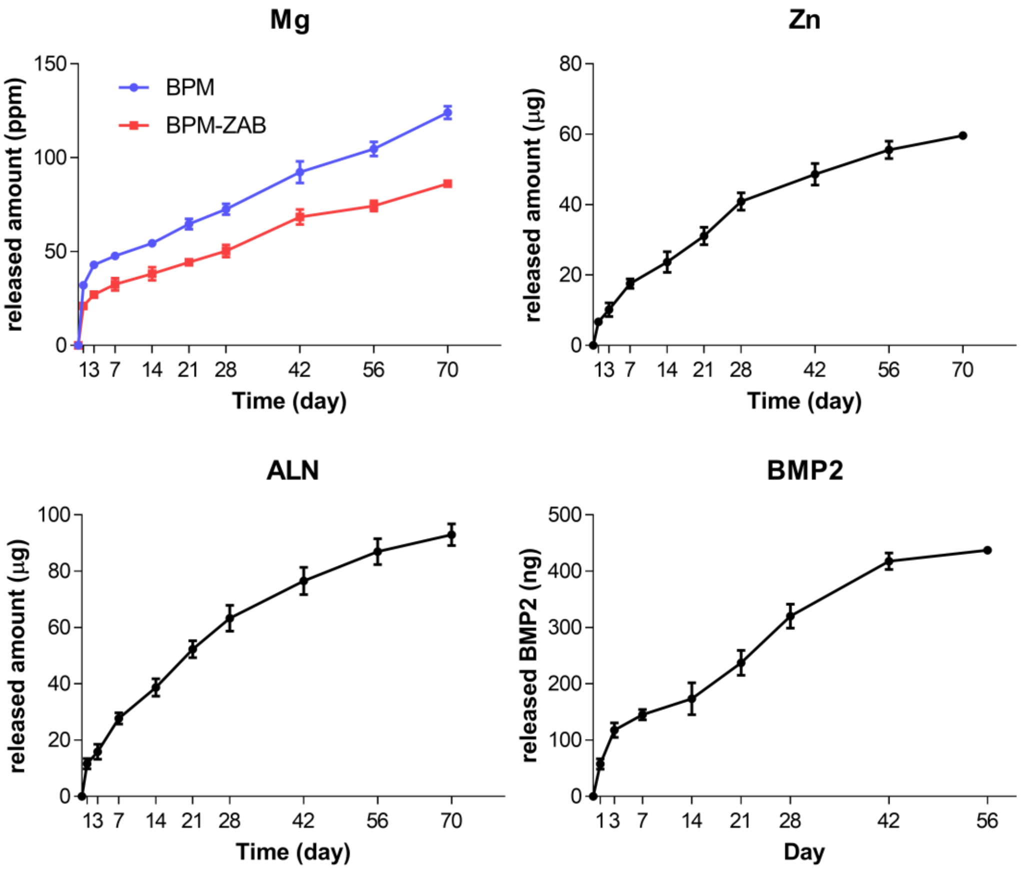

Figure S8. Cumulative release amount of each drug on the scaffolds. The degradation process was conducted for 70 days in PBS solution (n=3).

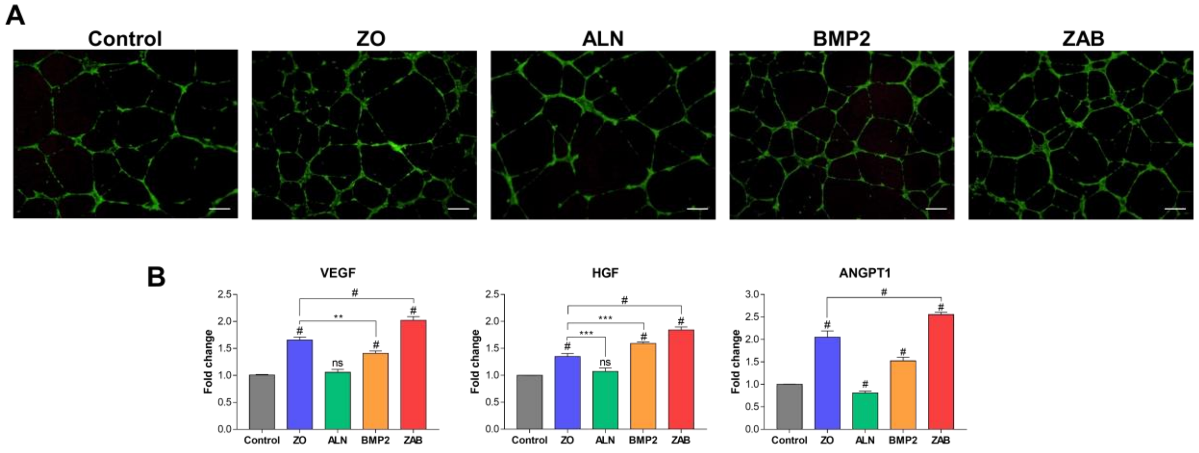

Figure S9. The angiogenic effects of ZAB without SNAP (n=3): (A) Tube forming assay; Calcein AM-stained images (scale bar = 100 μm) and (B) the expressions of the angiogenic factors with ZAB and its components. #$p < 0.0001$, ***$p < 0.001$, **$p < 0.01$, and *$p < 0.05$ indicate statistically significant differences, respectively.

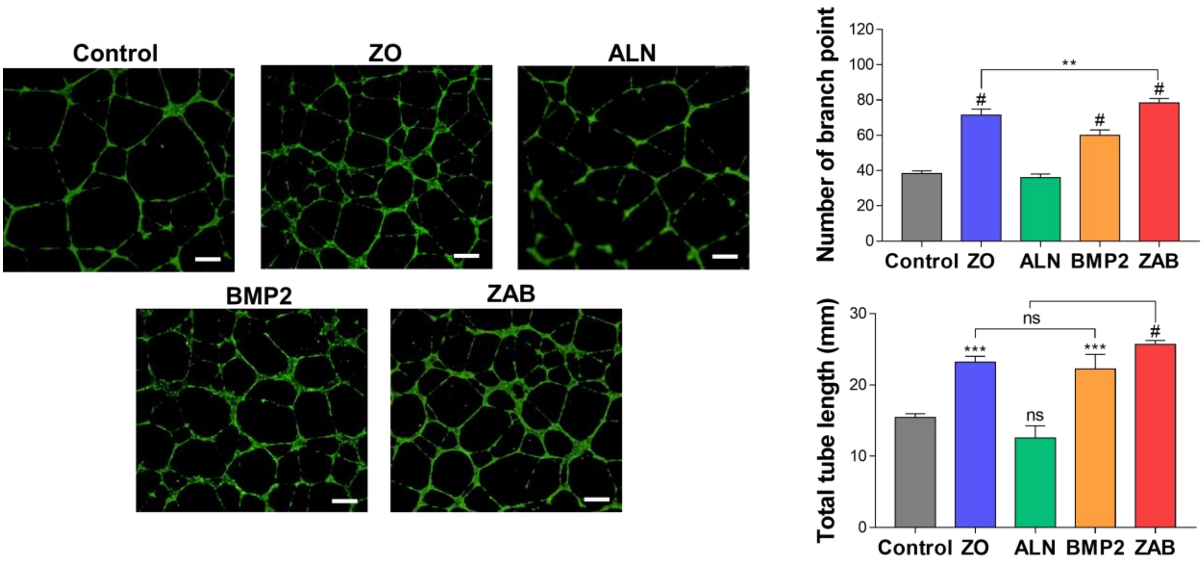

Figure S10. The angiogenic effects of ZAB with SNAP (n=3): (A) Tube forming assay; Calcein AM-stained images (scale bar = 100 μm) and (B) the expressions of the angiogenic factors with ZAB and its components. #*p* < 0.0001, ****p* < 0.001, ***p* < 0.01, and **p* < 0.05 indicate statistically significant differences, respectively.

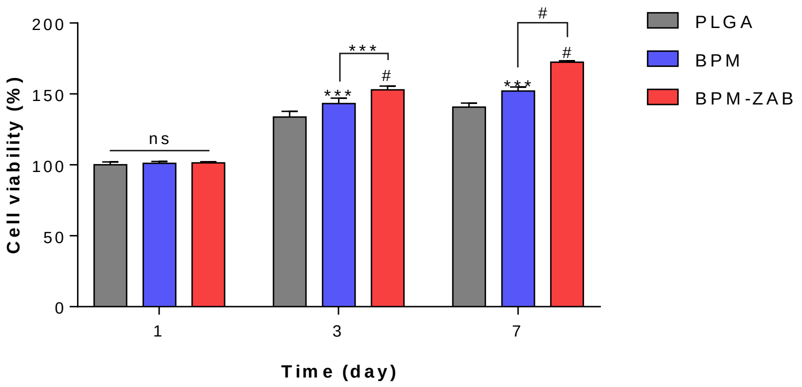

Figure S11. *In vitro* hBMSCs proliferation on the scaffolds after 1,3, and 7 days (n=3). The value was normalized by PLGA scaffold at day 1. #*p* < 0.0001, ****p* < 0.001, ***p* < 0.01, and **p* < 0.05 indicate statistically significant differences, respectively.

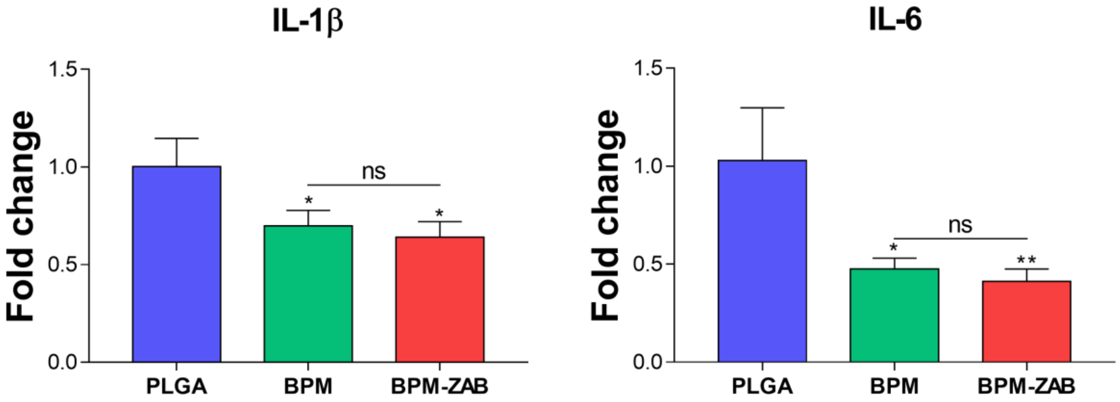

Figure S12. *In vitro* gene expression of inflammatory cytokines on the scaffolds at 3 days (n=3). The value was normalized by PLGA. #p < 0.0001, ***p < 0.001, **p < 0.01, and *p < 0.05 indicate statistically significant differences, respectively.

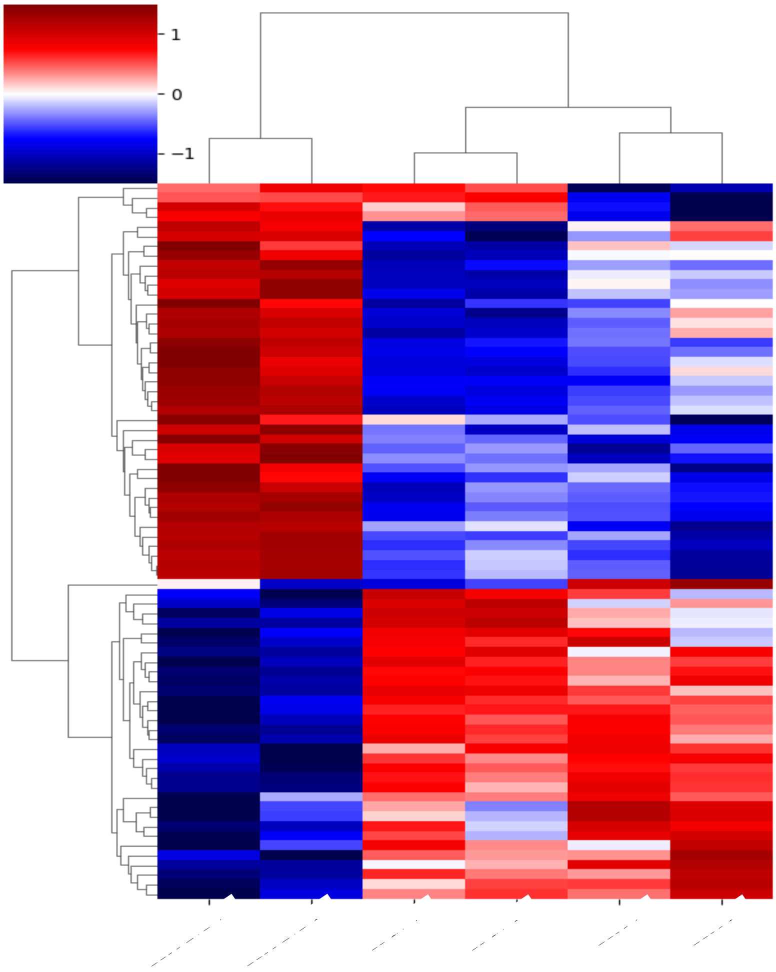

Figure S13. Total Heatmap and hierarchical clustering analysis of differentially expressed genes among various scaffold groups. The red indicates upregulated mRNA, while blue represents downregulated mRNA.

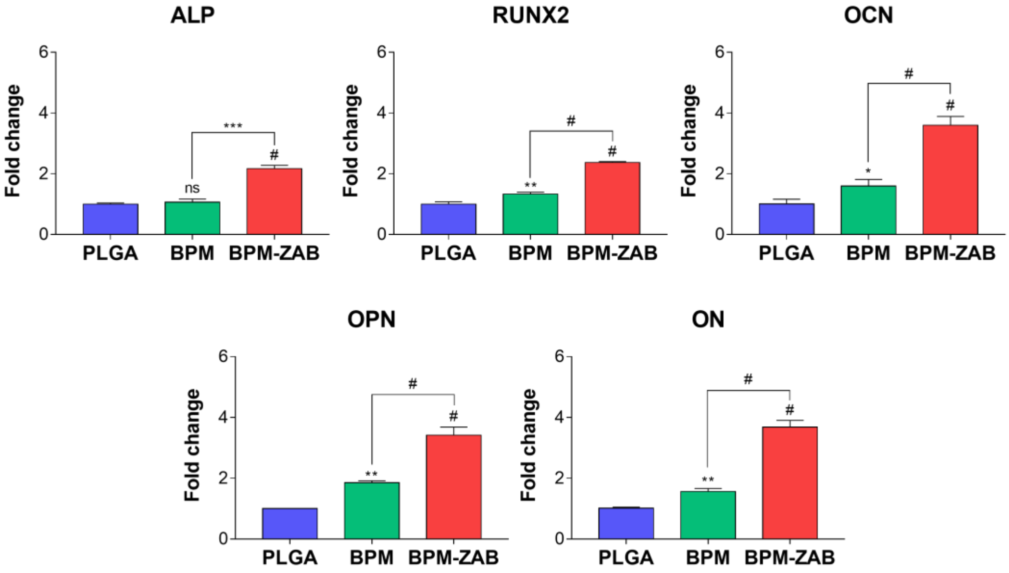

Figure S14. The mRNA expression levels related to osteogenesis quantified by RT-qPCR in 7 days of osteogenic differentiation; alkaline phosphatase (ALP), runt-related transcription factor 2 (RUNX2), osteocalcin (OCN), osteopontin (OPN), and osteonectin (ON). #*p* < 0.0001, *****p* < 0.001, ***p* < 0.01, and **p* < 0.05 indicate statistically significant differences, respectively.

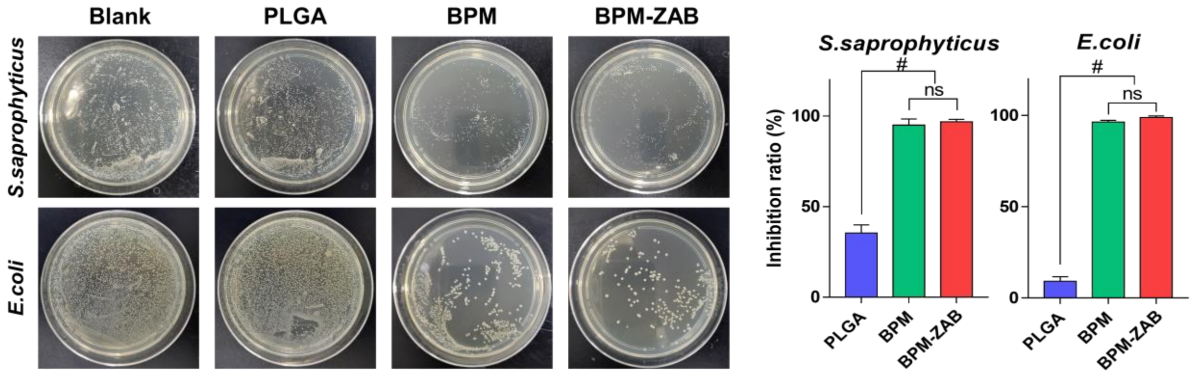

Figure S15. Anti-bacterial capacity of the scaffolds was evaluated with Gram-positive bacteria (*S. saprophyticus*) and Gram-negative bacteria (*E. coli*). Representative images of bacterial colonies formed by *E. coli* and *S. saprophyticus* on Luria-Bertani agar plates and quantified inhibition ratio. #*p* < 0.0001, ***p* < 0.001, **p* < 0.01, and *p* < 0.05 indicate statistically significant differences, respectively.

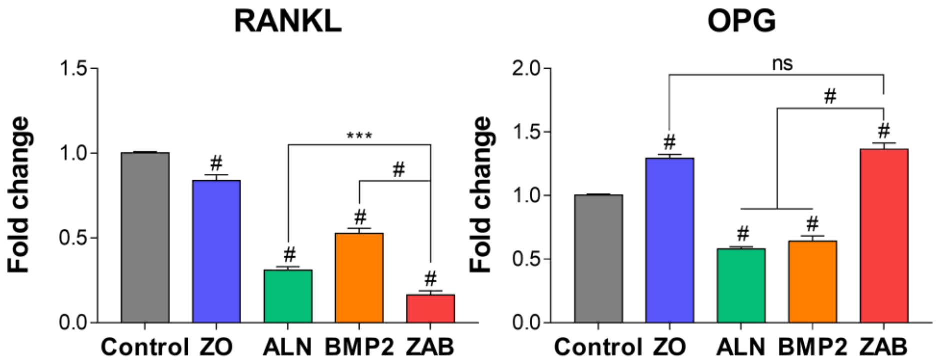

Figure S16. The gene expression of RANKL and OPG, respectively. #$p < 0.0001$, ***$p < 0.001$, **$p < 0.01$, and *$p < 0.05$ indicate statistically significant differences, respectively.

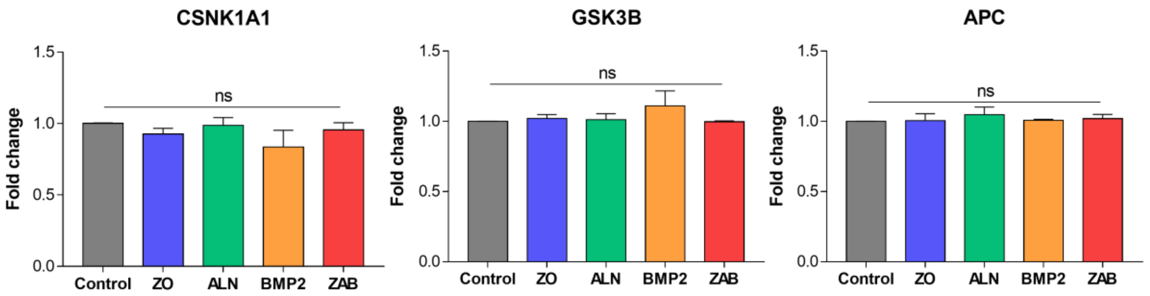

Figure S17. The Wnt/β-catenin pathway-related gene expressions (CSNK1A1, GSK3B, and APC). #$p < 0.0001$, ***$p < 0.001$, **$p < 0.01$, and *$p < 0.05$ indicate statistically significant differences, respectively.

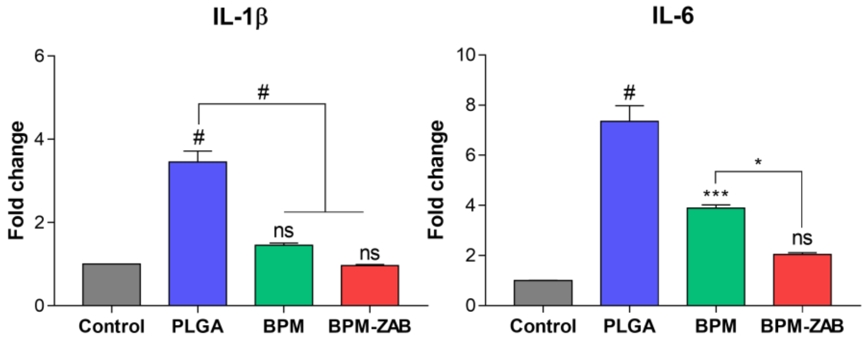

Figure S18. *In vivo* gene expression related to inflammation on non-OVX rat at 8 weeks (n=3). #$p < 0.0001$, ***$p < 0.001$, **$p < 0.01$, and *$p < 0.05$ indicate statistically significant differences, respectively.
